# DYADIC LONELINESS, AGE, AND COGNITIVE FUNCTIONING AMONG MID-LIFE AND OLDER BLACK COUPLES

**DOI:** 10.1093/geroni/igad104.0871

**Published:** 2023-12-21

**Authors:** Jeffrey E Stokes, Heather Farmer

**Affiliations:** University of Massachusetts Boston, Boston, Massachusetts, United States; University of Delaware, Newark, Delaware, United States

## Abstract

Cognitive health is a growing concern for the older Black population, whose risk for dementia is about twice that of White older adults. Moreover, close relationships, such as marriage, are critical for well-being in later life. Within older adults’ marriages, loneliness has been linked with individuals’ own and their partners’ mental, physical, and cognitive health. Yet little research has examined these relationships within the older Black population, and even less work has situated Black older adults within a relational, dyadic context. Lastly, research is needed on the circumstances under which partners’ loneliness may be influential for cognition, including potential variation by individuals’ age or their own level of cognitive functioning. This study used longitudinal dyadic data from the Health and Retirement Study (2010-2016; n=1,270 participants from 635 couples) from both partners in opposite-sex midlife and older married couples where at least one partner reported being Black or African American. Lagged dependent variable (LDV) structural equation modeling (SEM) addressed our research questions. Results indicated that (1) loneliness was not associated with participants’ own cognitive functioning 4-years later, yet (2) husbands’ loneliness was associated with worse cognitive functioning 4-years later for wives who had high baseline cognitive functioning themselves, and (3) wives’ loneliness was associated with worse cognitive functioning 4-years later only for oldest-old husbands. Findings indicate that loneliness has dyadic consequences for cognitive functioning among older Black couples, but that context is crucial for determining which older partners are at greatest risk of experiencing harmful cognitive repercussions from a partner’s loneliness.

